# Supplementary figures and images for: Radiotherapy-Induced Changes in the Systemic Immune and Inflammation Parameters of Head and Neck Cancer Patients
Source: Cancers (Basel). 2019 Sep 6;11(9):1324. doi: 10.3390/cancers11091324 (PMC6770727; doi:10.3390/cancers11091324)

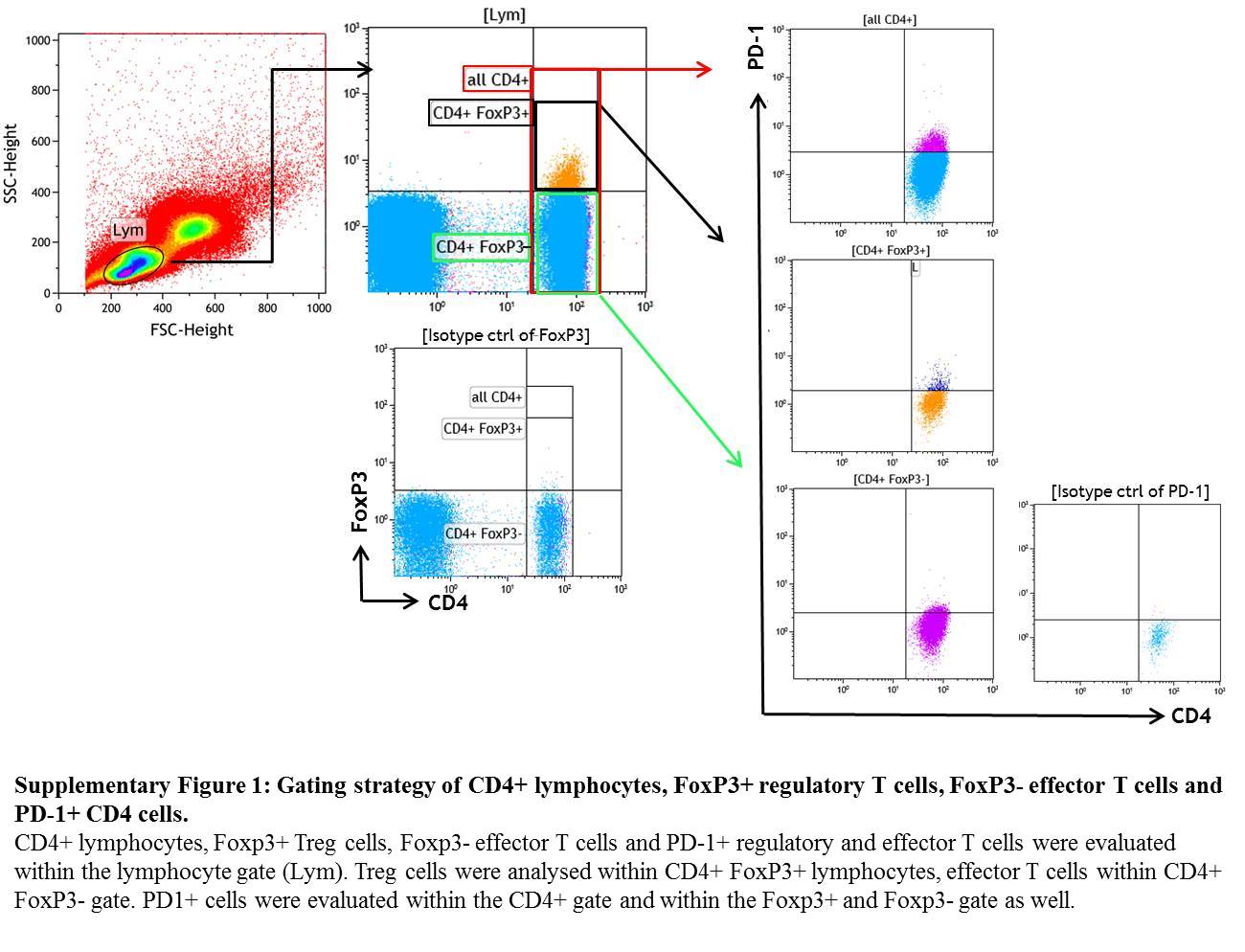

Supplement: Supplementary file 1 [file cancers-11-01324-s001.zip › Supplementary Figures feltöltésre/Supplementary Figure 1.jpg]

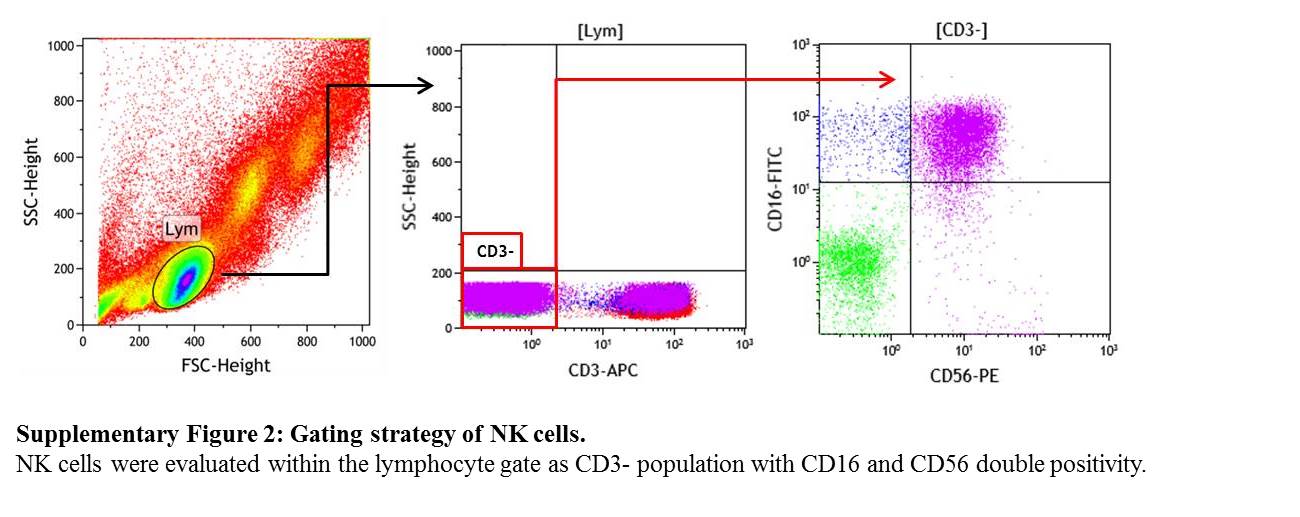

Supplement: Supplementary file 1 [file cancers-11-01324-s001.zip › Supplementary Figures feltöltésre/Supplementary figure 2.jpg]

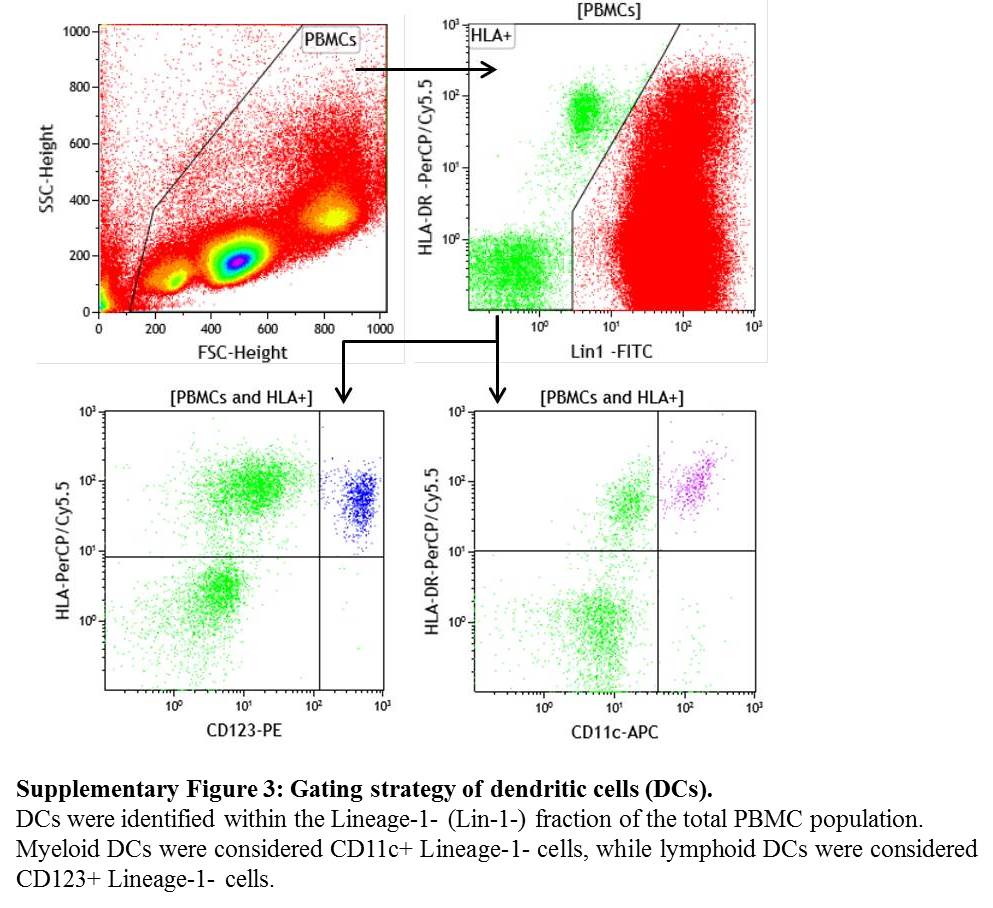

Supplement: Supplementary file 1 [file cancers-11-01324-s001.zip › Supplementary Figures feltöltésre/Supplementary figure 3.jpg]

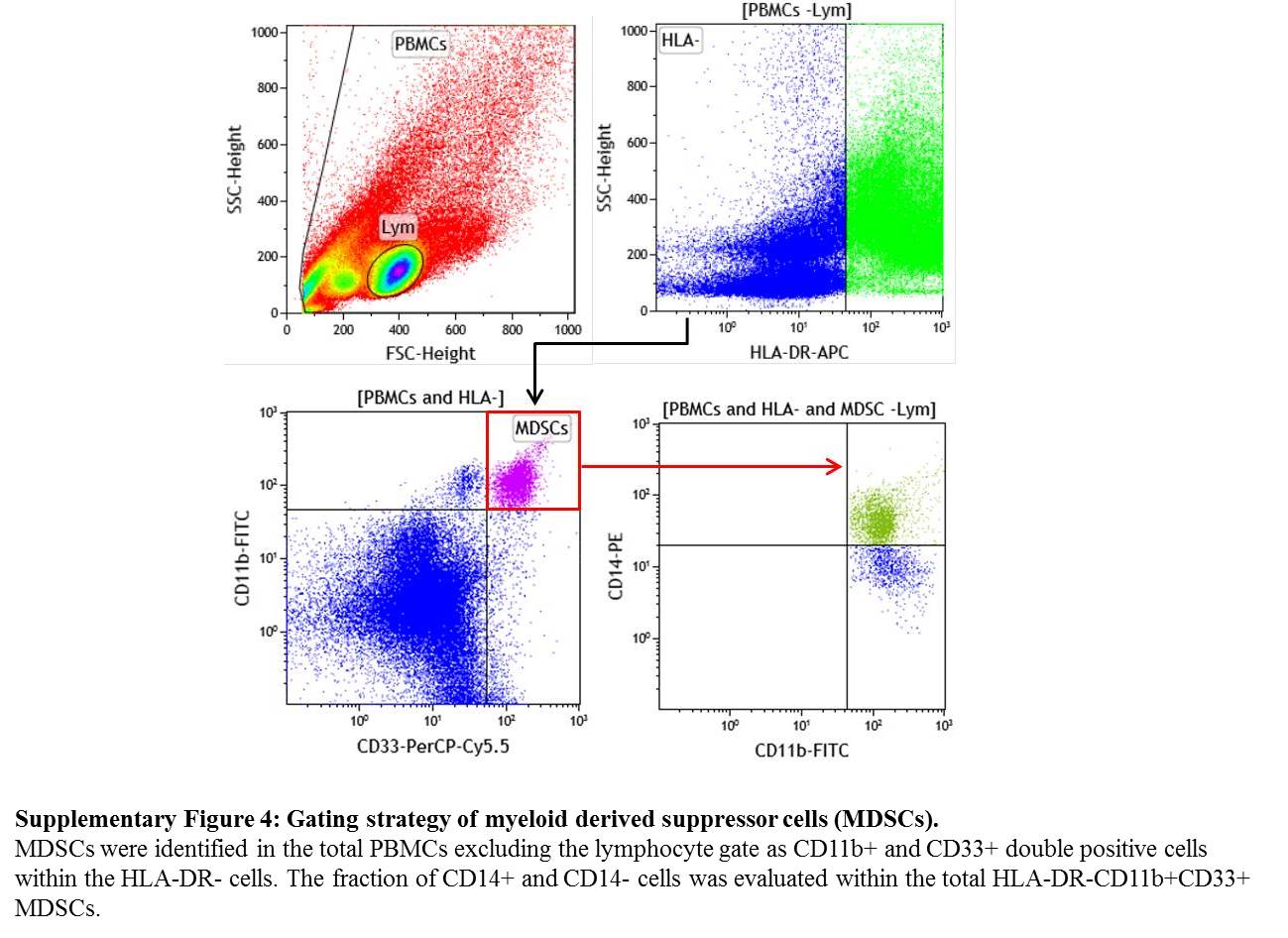

Supplement: Supplementary file 1 [file cancers-11-01324-s001.zip › Supplementary Figures feltöltésre/Supplementary figure 4.jpg]

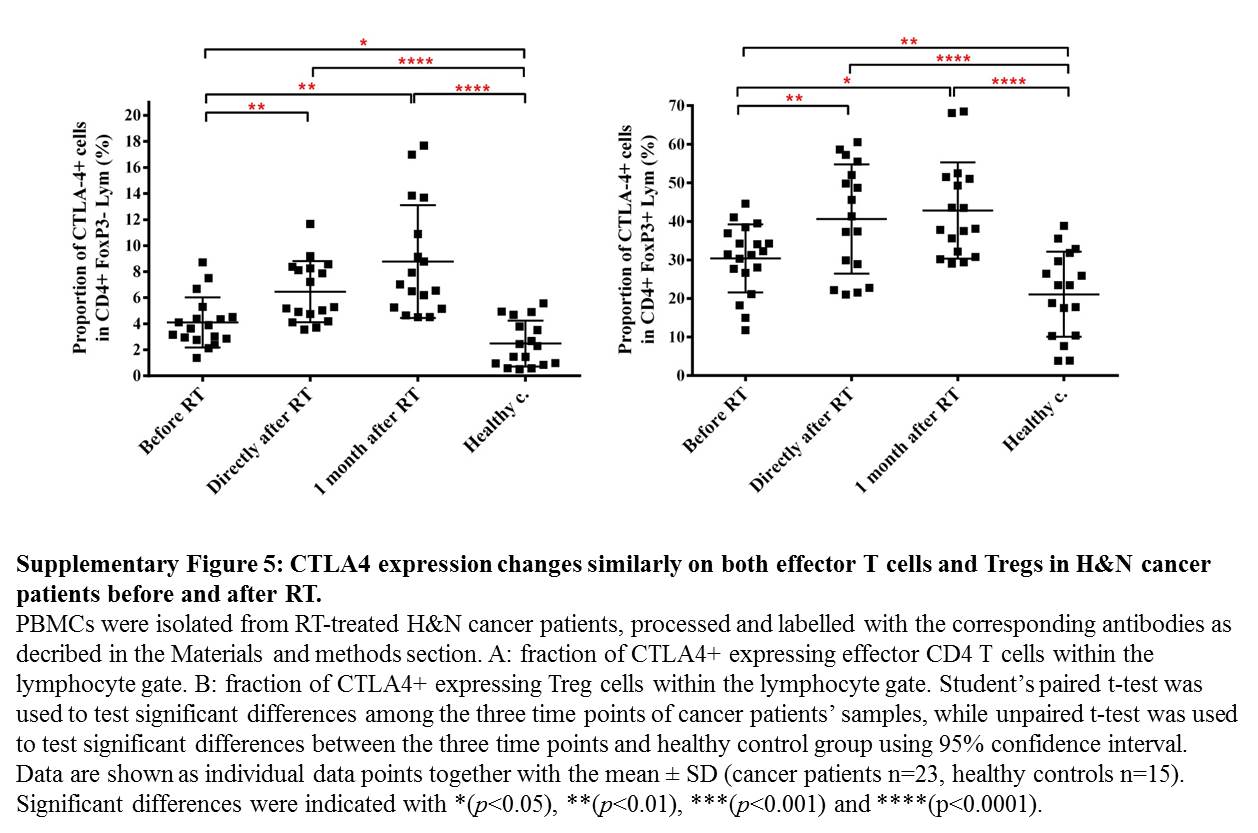

Supplement: Supplementary file 1 [file cancers-11-01324-s001.zip › Supplementary Figures feltöltésre/Supplementary figure 5.jpg]

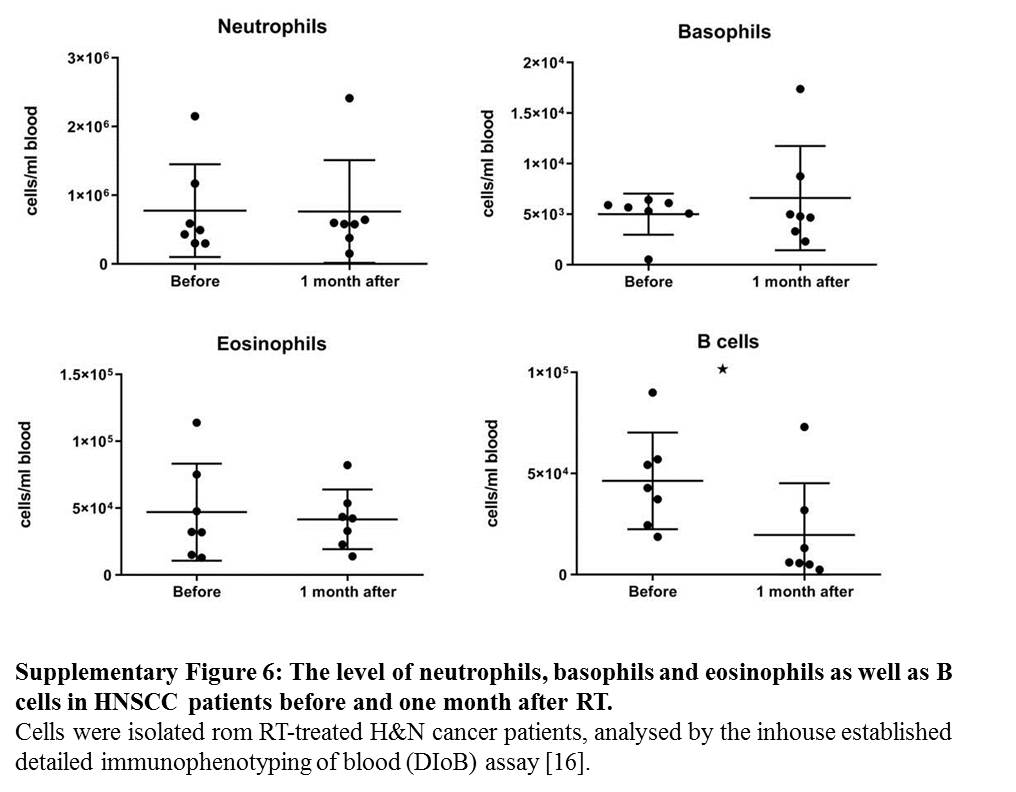

Supplement: Supplementary file 1 [file cancers-11-01324-s001.zip › Supplementary Figures feltöltésre/Supplementary Figure 6.jpg]
